# Cannabis use in patients with early psychosis is associated with alterations in putamen and thalamic shape

**DOI:** 10.1002/hbm.25131

**Published:** 2020-07-20

**Authors:** Musa Sami, James H. Cole, Matthew J. Kempton, Luciano Annibale, Debasis Das, Marlene Kelbrick, Savitha Eranti, Tracy Collier, Chidimma Onyejiaka, Aisling O'Neill, David J. Lythgoe, Philip McGuire, Steve C. R. Williams, Sagnik Bhattacharyya

**Affiliations:** ^1^ Institute of Psychiatry Psychology and Neurosciences King's College London London UK; ^2^ Leicestershire Partnership NHS Trust London UK; ^3^ Northamptonshire Healthcare NHS Foundation Trust Kettering UK; ^4^ East London Foundation Trust London UK; ^5^ Institute of Psychiatry Psychology and Neuroscience London UK

**Keywords:** cannabis, psychotic disorders, shape analysis, subcortical shape

## Abstract

Around half of patients with early psychosis have a history of cannabis use. We aimed to determine if there are neurobiological differences in these the subgroups of persons with psychosis with and without a history of cannabis use. We expected to see regional deflations in hippocampus as a neurotoxic effect and regional inflations in striatal regions implicated in addictive processes. Volumetric, T1w MRIs were acquired from people with a diagnosis psychosis with (PwP + C = 28) or without (PwP − C = 26) a history of cannabis use; and Controls with (C + C = 16) or without (C − C = 22) cannabis use. We undertook vertex‐based shape analysis of the brainstem, amygdala, hippocampus, globus pallidus, nucleus accumbens, caudate, putamen, thalamus using FSL FIRST. Clusters were defined through Threshold Free Cluster Enhancement and Family Wise Error was set at *p* < .05. We adjusted analyses for age, sex, tobacco and alcohol use. The putamen (bilaterally) and the right thalamus showed regional enlargement in PwP + C versus PwP − C. There were no areas of regional deflation. There were no significant differences between C + C and C − C. Cannabis use in participants with psychosis is associated with morphological alterations in subcortical structures. Putamen and thalamic enlargement may be related to compulsivity in patients with a history of cannabis use.

## Graphical abstract

Cannabis is often associated with the early course of psychotic disorders. The EfCIP study looked at four groups: People with psychosis with cannabis use, people with psychosis without cannabis use, controls with cannabis use and controls without cannabis use. We observe change in subcortical morphology in persons with psychosis with cannabis use versus persons with psychosis without cannabis use in the putamen and thalamus which may be due to compulsive use in this group.

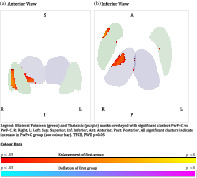

## 1 INTRODUCTION

The use of cannabis by people with a diagnosis of psychosis (PwP) is associated with worse clinical outcomes. PwP who use cannabis have an earlier age of psychosis onset (Large, Sharma, Compton, Slade, & Nielssen, 2011), poorer functioning as indexed by the Global Assessment of Functioning (GAF; Seddon et al., 2015), increased healthcare utilisation (Manrique‐Garcia et al., 2014; Patel et al., 2016), and increased rates of relapse (Schoeler et al., 2016). However there is also evidence that PwP who use cannabis have less severe impairments in cognitive function particularly in the early phase of psychosis (Schoeler, Kambeitz, & Bhattacharyya, 2015; Stirling, Lewis, Hopkins, & White, 2005; Yucel et al., 2012) and display fewer neurological soft signs (Bersani, Orlandi, Gherardelli, & Pancheri, 2002; Mallet, Ramoz, Le Strat, Gorwood, & Dubertret, 2017; Mhalla et al., 2017; Ruiz‐Veguilla et al., 2009). The neurobiological basis of these differences is unclear. One way of addressing this issue is to compare brain anatomy in PwP with or without a history of cannabis use.

A systematic review (Sami & Bhattacharyya, 2018) demonstrated that after 5 years of psychosis onset, cannabis using PwP exhibited a greater degree of grey matter reduction (Rais et al., 2008) and cortical thinning (Habets, Marcelis, Gronenschild, Drukker, & Van Os, 2011; Rais et al., 2010) with evidence of longitudinal deterioration (Rais et al., 2008; Rais et al., 2010) than PwP who did not use cannabis. It is likely that this evidences either neurotoxic effects of ongoing substance use or alternatively a distinct trajectory of the psychosis.

However, in the early stages of cannabis use in psychosis (≤5 years after illness onset), the morphological characterisation has been less clear. Some studies have shown relative enlargement of hippocampal (Cunha et al., 2013), putamen (Koenders et al., 2015) and cortical regions (Cunha et al., 2013; Schnell, Kleiman, Gouzoulis‐Mayfrank, Daumann, & Becker, 2012) and localised increase in the corpus collosum (Malchow et al., 2013) in PwP with cannabis use compared to those who do not use the drug. Others have, however, shown reductions in volume of mediotemporal, striatal, (James et al., 2011), thalamic (Kumra et al., 2012), cerebellar (Cohen et al., 2012; James et al., 2011), and cortical regions (James et al., 2011; Rapp et al., 2013; Szeszko et al., 2007) in PwP with cannabis use compared to PwP who do not use cannabis. Other studies in early psychosis have found no volumetric differences between PwP who do and not use cannabis (Bangalore et al., 2008; Cahn et al., 2004; Haller et al., 2013).

Taken together, although there is well‐replicated evidence for grey matter reduction of cannabis use in people with longer term psychosis that is, more than 5 years (Habets et al., 2011; Rais et al., 2008; Rais et al., 2010) the literature is unclear about differences in brain morphology related to cannabis use in individuals in the earlier phase of psychosis (i.e., less than 5 years). Possible reasons for variation in results between studies may be due to differences in regions of interest (brain volume, grey matter, cerebellum, cortical thickness, subcortical structures), differences in sample composition (enduring psychotic disorder, schizophrenia spectrum disorder, early psychosis), medication (whether medication status is accounted for), definition of cannabis use consumption (ever use or misuse/dependence) and differences in which confounders were adjusted for (tobacco and alcohol). For a full review of these differences see (Sami & Bhattacharyya, 2018). Taken together it was suggested that in the early stages of psychosis, cumulative exposure to cannabis has been shorter than in later stages of psychosis and hence neurobiological differences between cannabis using and cannabis‐free PwP may be more subtle (Sami & Bhattacharyya, 2018). Therefore, a more sensitive technique may be necessary to detect any group differences.

Most studies to date have undertaken volumetric analysis of structures which provide a global measure of a structure whilst few studies have looked at shape analysis. Although volume changes indicate global change in morphology. Shape analysis allows for the detailed examination of morphometric alterations. Hence the two analyses may provide complementary information. Analysis of shape deformation parameterises high‐dimensional changes in complex structures and hence may be more sensitive to regional effects (Gerig, Styner, Shenton, & Lieberman, 2001). For example, the thalamus includes 50–60 different nuclei and the amygdala and hippocampi are similarly amalgamations of distinct substructures (Fanselow & Dong, 2010; Herrero, Barcia, & Navarro, 2002; Janak & Tye, 2015), hence, perturbation of any substructure may distort shape with limited appreciable effect on total volume. Both psychosis and cannabis use, studied on their own, have been shown to be associated with subcortical shape changes. In psychotic disorders there are known alterations in the shape of caudate (Scanlon et al., 2014), hippocampus (Sauras et al., 2017) and thalamus (Smith et al., 2011) compared to controls. Cannabis use is associated with enlargement of the nucleus accumbens and amygdala in recreational users (Gilman et al., 2014) and regional hippocampal deflation in cannabis dependent PwP (Chye et al., 2019a).

To date, no study has examined shape changes in the early phase of psychosis and cannabis use. Three studies have looked at the shape of subcortical structures in individuals with chronic psychosis with and without cannabis use. Solowij et al. (2013) examined chronic patients with schizophrenia with average duration of psychosis 19.1 years. The study demonstrated regional deflations in hippocampus in cannabis users compared to non‐users but no difference after False Discovery Rate correction. Smith et al. (2014) and Smith et al. (2015) undertook two analyses in a larger sample, finding cannabis related shape deformations in hippocampus, striatum, globus pallidus and thalamus which were associated with cognitive performance. These patients were less chronic, albeit average duration of psychosis was 6.5 years. As shown in our previous Systematic Review (Sami & Bhattacharyya, 2018) and discussed above, 5 years seems to be a key period where morphological changes have not been clear, and this has not been undertaken using shape analyses to date. Study of these substrates in an earlier sample may help more clearly delineate early markers of pathology distinct from the longer term effects of psychosis and substance use.

Hence, the aim of the present study was to examine the association between cannabis use and the shape of subcortical structures in individuals in the early phase of psychosis, focusing on the major deep grey matter regions. After review of previous studies in the field (Sami & Bhattacharyya, 2018) we also included controls with or without a history of cannabis use. A number of subcortical structures have been implicated in cannabis and psychosis as outlined above so we explored this in the main subcortical structures. In line with prior literature, reviewed above (Chye et al., 2019a; Koenders et al., 2015; Smith et al., 2015), we had expected to see regional deflations in the hippocampus in cannabis using participants with psychosis compared to non‐cannabis using participants with psychosis as a neurotoxic effect of cannabis; and regional inflations in shape of striatal regions which are involved in addiction circuitry. We also tested whether these differences would be evident between controls with and without a history of cannabis use.

## 2 METHODS

### 2.1 Sample

All data were acquired from the EfCiP study (the Effect of Cannabis in Psychosis (London‐Stanmore REC 17/LO/0577) which recruited four groups: Early Psychosis individuals with a history of cannabis use (PwP + C; *n* = 28), Early Psychosis individuals without a cannabis use history (PwP − C; *n* = 26), controls with a history of cannabis use (C + C; *n* = 16) and controls without such a history (C − C; *n* = 22).

Early Psychosis was defined as first treatment of psychosis within the last 5 years. One patient under an Early Intervention in Psychosis team had experienced a psychotic episode 20 years ago at the age of 12 and had been asymptomatic off‐treatment until re‐presenting in his 30 s. Cannabis use was defined as use of cannabis ≥20 times in lifetime but we preferentially recruited for heavy cannabis use in both PwP and controls. We excluded participants with organic psychosis and those with substance intoxication on the study day. PwP were recruited through clinical teams from 16 NHS trusts across England through the National Institute of Health Research (UK) Clinical Research Networks.

### 2.2 Study day

Participants attended a study day including psychiatric and drug interview schedules and Magnetic Resonance Imaging. Each participant was assessed with the Structured Clinical Interview for DSM‐IV (SCID‐IV; First et al., 1995), GAF(First et al., 1995), and Positive and Negative Syndrome Scale (PANSS)(Kay, Fiszbein, & Opler, 1987). National Assessment of Reading Test (NART) was used to index Full Scale Intelligence Quotient using a recently restandardised calculation in British adults (Bright, Hale, Gooch, Myhill, & van der Linde, 2018). Since NART is not valid in Learning Disability where participants had a previously diagnosed mild Learning Disability they were assigned an IQ score of 65. Participants were administered the Fagerstrom test for Nicotine Dependence (Heatherton, Kozlowski, Frecker, & Fagerström, 1991), the Alcohol Use Disorders Identification Test (AUDIT; Babor, Higgins‐Biddle, Saunders, & Monteiro, 2001), the Severity Dependence Scale (SDS; Gossop et al., 1995) in relation to the last month's cannabis use, the TimeLine FollowBack questionnaire (Sobell & Sobell, 1996) and a modified version of the Cannabis Experiences Questionnaire we have previously used to index quantity of cannabis and other drug use (Barkus, Stirling, Hopkins, & Lewis, 2006; Schoeler et al., 2016). Chlorpromazine equivalents were calculated from the Maudsley Guidelines 12th Edition, or where not available therein, from Gardener et al. (Gardner, Murphy, O'Donnell, Centorrino, & Baldessarini, 2010; Taylor, Paton, & Kapur, 2016). As NART was added after the 20th participant, these data for participants prior to this were collected subsequently by telephone, with NART assumed to be stable over time.

### 2.3 Image acquisition

Data were acquired using a 32‐channel head coil (Nova Medical, Wilmington, MA) on a General Electric Healthcare MR750 (Chicago, IL) 3 Tesla system. After a 3‐plane localizer for orientation and an ASSET calibration, T1 weighted volumetric images were acquired using the sagittal ADNI Go Inversion Recovery Spoiled Gradient Echo (IR‐SPGR) sequence with 196 1.2 mm thick slices and an in‐plane matrix size of 256 x 256 (1.05 mm × 1.05 mm) (TR/TI/TE: 7.312 ms/400 ms/3.016 ms, flip angle: 11°). The field of view was placed to avoid nose wrap. Total acquisition time was 5 min 37 seconds.

### 2.4 Statistical analysis

Demographic, clinical and cannabis use data were investigated using t‐tests for continuous variables and chi‐squared tests for categorical variables. Mann–Whitney *U* test was used for skewed data and median and interquartile Range (IQR) reported. Analysis was undertaken using the SPSS version 25. Imaging data analysis was conducted as described below.

### 2.5 Structural MRI processing

All T1 weighted images were inspected for image quality and evidence of motion and MRI artefact. We undertook Shape Analysis using an automated vertex‐based approach using the FMRIB laboratory's Integrated Registration & Segmentation Tool (FSL FIRST) (https://fsl.fmrib.ox.ac.uk/fsl/fslwiki/FIRST). Briefly, this relies on Active Appearance Modelling (AAM) algorithm (Cootes, Edwards, & Taylor, 1998) which is an optimised shape recognition technique whereby the algorithm is trained using an iterative procedure to minimise residuals between estimated shape and the gold standard, incorporating both shape and intensity information of the image. For the purpose of FSL FIRST this has been trained on a manually labelled dataset of 336 brain images of 15 structures in both health and disease. The FIRST toolkit undertakes AAM on the provided data by modelling shape of subcortical structures such that the models from the training set act as a prior probability in a Bayesian framework (Patenaude, Smith, Kennedy, & Jenkinson, 2011). Firstly, affine transformation was undertaken to register scans to standard Montreal Neurological Institute (MNI) space using the 1mm^3^ isotropic MNI 152 template. Next, 15 subcortical structures (i.e., seven bilateral structures left and right: amygdala, caudate, hippocampus, nucleus accumbens, pallidum, putamen, thalamus and brainstem) were segmented. Segmentations were manually checked for errors. One scan (PwP + C) was rejected due to registration failure and was excluded from further analysis.

Having generated vertex‐based segmentations—all subcortical structure files were concatenated into a 4D volume such that corresponding vertices across subjects were projected onto the average shape of the cohort. Each image represented a subject and each projected vertex in the image represented the perpendicular distance from the corresponding point on the average surface.

For shape analysis we undertook group‐wise statistical testing of surface vertices of subcortical structures using FSL Randomise—a permutation based analysis, a non‐parametric method, for inferential testing (Winkler, Ridgway, Webster, Smith, & Nichols, 2014). Analysis was undertaken using 10,000 permutations for each subcortical structure. Using General Linear Models, we tested group effects regressing out the effects of demeaned covariates: age, sex, AUDIT score (for alcohol use) and Fagerstrom score (for tobacco use) as covariates of no interest. Three contrasts were tested in line with our hypotheses: PwP + C versus PwP − C; all PwP versus all controls; C + C versus C − C. Clusters were defined through Threshold Free Cluster Enhancement, a cluster‐based method of multiple correction using neighbouring vertex information without prior arbitrary definition of a cluster‐forming threshold (Smith & Nichols, 2009) and family wise error was set at *p* < .05.

In a further exploratory analysis we undertook a volume‐based analysis of all subcortical structures. From each segmentation we extracted the volume of each structure for each participant. We tested the same three contrasts between groups for each structure using t‐tests with Bootstrapped Bias‐Corrected accelerated (BCa) 95% confidence intervals (1,000 samples). We report results as significant at *p* < .05. To test whether significant differences would survive multiple comparison correction we undertook false discovery rate correction for the volume of each shape within each contrast.

In a secondary analysis (as informed by the results of the primary analysis) we wished to determine whether the findings seen in PwP + C versus PwP − C were similar to those seen in Cannabis Dependence. We consequently undertook the same analyses which have been described above for a further contrast: those with lifetime Cannabis Dependence (as diagnosed by the SICD) versus those without lifetime Cannabis Dependence across the whole sample. These are presented in the main manuscript (for surface analyses) and Supporting Information (for volumetric analyses) alongside the main findings.

## 3 RESULTS

*Demographics*: Data was available for 54 PwP and 38 controls. Baseline demographic and clinical data can be seen in Table 1. Of note there were no clinically significant differences between PwP + C and PwP − C groups across all clinical variables: proportion with schizophrenia spectrum disorder diagnosis, total PANSS scores and all subscales, GAF scores, days spent in hospital, age at onset and chlorpromazine equivalent antipsychotic dose. As expected, there were increased Fagerstrom and AUDIT scores in the PWP + C group indexing increased tobacco and alcohol use in PwP with a history of cannabis use. PwP had lower estimated IQ than controls.

**TABLE 1:** Baseline characteristics between groups

	*PwP + C (n = 28)*		*PwP* − *C (n = 26)*		*C + C (n = 16)*		*C + C (n = 22)*	
	Mean	*SD*	Mean	*SD*	Mean	*SD*	Mean	*SD*
Male sex (proportion)	78.6%		65.4%		62.5%		50%	
Age	25.49	*3.93*	26.73	*5.15*	27.11	*5.95*	28.16	*5.29*
Fagerstrom^a^ ^,^ ^b^ (nicotine dependence)	2.68	*2.44*	0.62	*1.50*	0.75	*1.73*	0.00	*0.00*
AUDIT^b^ ^,^ ^c^ (alcohol use disorders)	8.82	*5.40*	3.42	*4.83*	7.75	*6.43*	3.59	*2.99*
Days in hospital	53.57	*75.26*	43.54	*61.60*				
Chlorpromazine equivalent medication dose	189.20	*177.34*	188.02	*173.25*				
Age of psychosis onset	23.34	*4.03*	23.66	*5.40*				
Schizophrenia spectrum (proportion)	78.6%		76.9%					
PANSS positive^a^	12.36	*5.40*	11.50	*5.37*	7.19	*0.54*	7.09	*0.43*
PANSS negative^a^ ^,^ ^c^	14.18	*7.24*	14.65	*6.58*	8.13	*1.59*	7.14	*0.64*
PANSS general^a^ ^,^ ^c^	27.50	*9.12*	27.00	*8.90*	19.25	*3.75*	16.82	*1.74*
PANSS total^a^ ^,^ ^c^	54.04	*18.32*	53.15	*17.45*	34.56	*5.38*	31.05	*2.13*
GAF^a^ ^,^ ^c^	69.89	*8.95*	72.65	*11.29*	89.25	*4.93*	93.32	*2.64*
Estimated IQ^a^	99.95	*12.74*	99.61	*14.35*	110.28	*6.93*	110.23	*7.98*
Time to use 3.5 g of cannabis (days)	9.89	*11.50*			7.96	*9.27*		
Age of first use	16.19	*2.48*			16.00	*2.50*		
THC in urine drug sample (proportion)	42.8%				43.8%			
Days since last use	Mdn: 62.5	*IQR*:*1–380*			Mdn: 7	*IQR*:*1–1*,*402*		
Cannabis use disorder (proportion)^d^	78.6%				50.0%			
Cannabis dependence (proportion)	60.7%				37.5%			

*Note:* Significance determined using *t*‐tests for continuous variables, Mann–Whitney *U* test for skewed data (median and IQR reported); chi‐squared for proportions: *p* < .05.

Abbreviations: AUDIT, alcohol use disorder identification test; C + C Controls with history of cannabis use; C − C: Controls without history of cannabis use; GAF, global assessment of functioning; IQ, intelligence quotient; IQR, interquartile range; Mdn, median; PANSS, positive and negative syndrome scale; SD, standard deviations; THC, tetrahydrocannabinol; PwP + C, early Psychosis with history of cannabis use; PwP − C, early Psychosis without history of cannabis use.

^a^ Significant difference patients versus controls, *p* < .05.

^b^ Significant difference PwP + C versus PwP − C, *p* < .05.

^c^ Significant difference C + C versus C − C, *p* < .05.

^d^ Trend level difference C + C versus C − C, *p* = .0503.

There was no discernible difference in cannabis use parameters between patient and control cannabis using groups (PwP + C vs. C + C) in terms of extent of use, age of first use, current use and days since last use (see Table 1). Proportionally more PwP + C met threshold for a lifetime diagnosis of cannabis use disorder (SCID IV abuse or dependence): there was a trend to increases cannabis misuse in PwP + C versus C + C (*p* = .0503).

**FIGURE 1:**
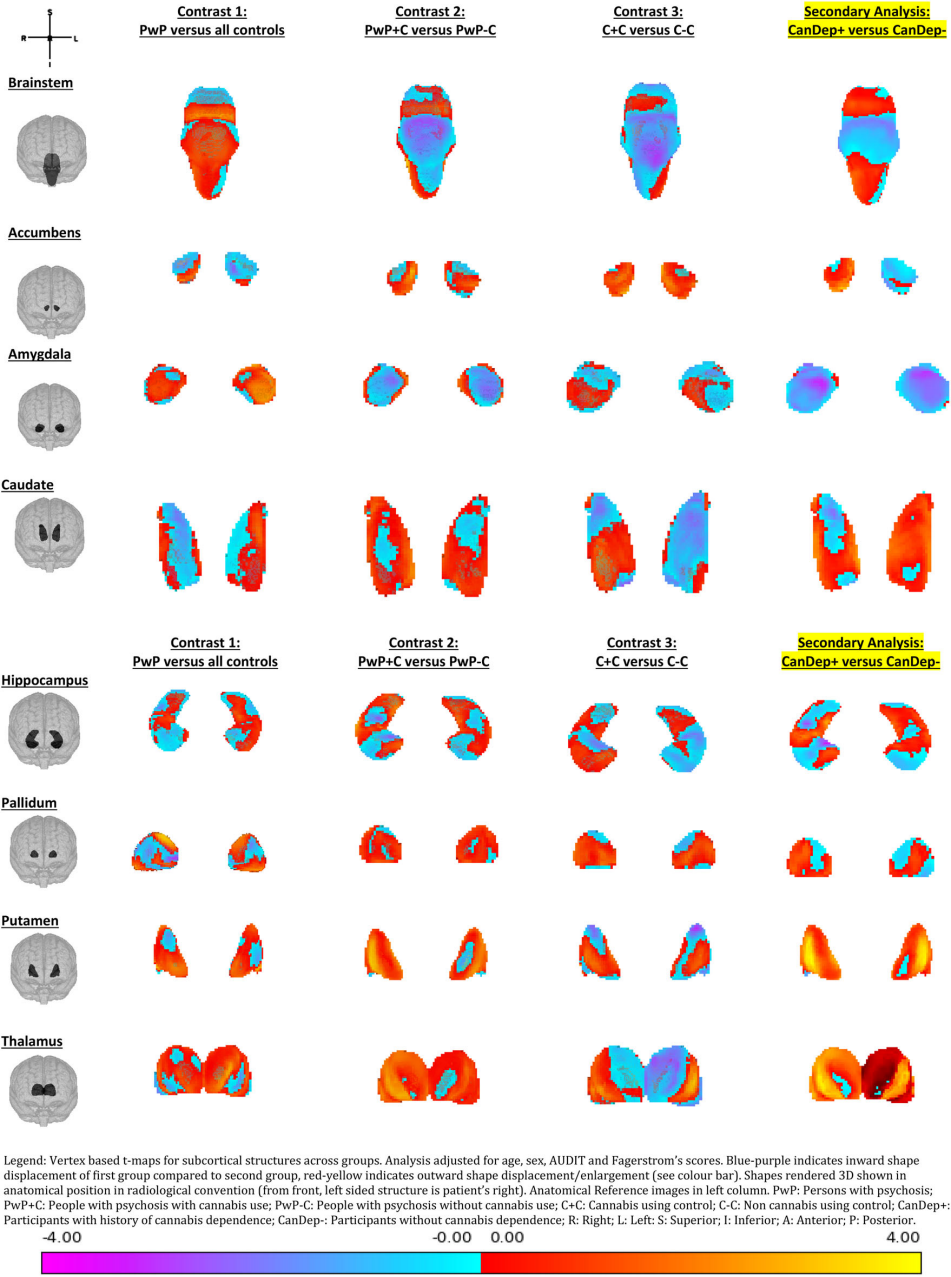
Vertex based t‐maps for subcortical structures across groups. Analysis adjusted for age, sex, AUDIT, and Fagerstrom's scores. Blue‐purple indicates inward shape displacement of first group compared to second group, red‐yellow indicates outward shape displacement/enlargement (see colour bar). Shapes rendered 3D shown in anatomical position in radiological convention (from front, left sided structure is patient's right). Anatomical Reference images in left column: A, anterior; I, inferior; L, left; P, posterior; R, right; S, superior

**FIGURE 2:**
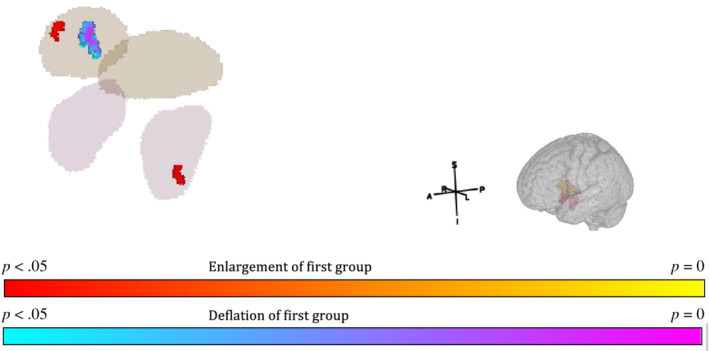
Bilateral Pallidum (brown) and Amygdala (purple) masks overlayed with significant clusters for all patients versus all controls. A, anterior; I, inferior; L, left; P, posterior; R, right; S, superior. Red‐yellow cluster indicates significant increase in patient group, blue‐purple cluster indicates significant decrease in patient group (see colour bar). TFCE, FWE *p* < .05

**FIGURE 3:**
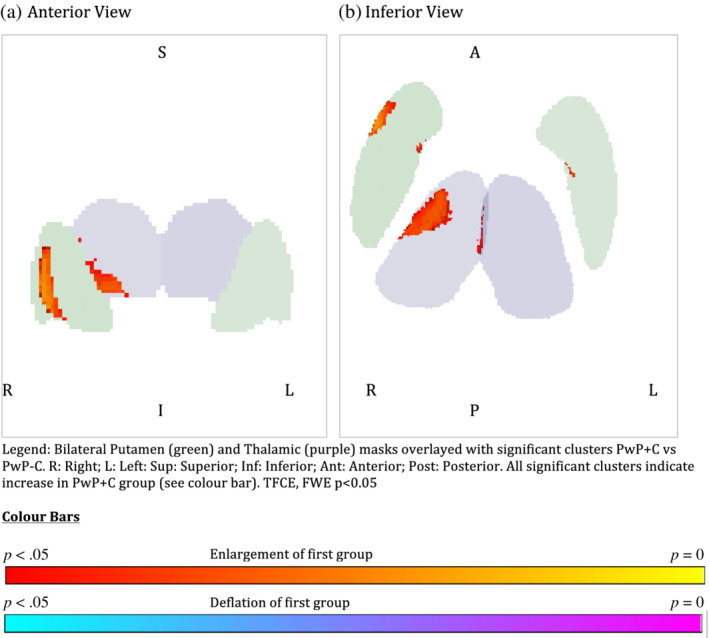
Legend: Bilateral Putamen (green) and Thalamic (purple) masks overlayed with significant clusters PwP + C versus PWP‐C. A, anterior; I, inferior; L, left; P, posterior; R, right; S, superior. All significant clusters indicate increase in PwP + C group (see colour bar). TFCE, FWE *p* < .05

*Shape*: Uncorrected T‐maps for the three contrasts can be seen in Figure 1. After TFCE correction and family wise error *p* < .05 correction, the following differences were noted: *PwP* versus *controls*: There were small clusters of enlargements in the right pallidum and left amygdala for PwP compared to controls (voxels ≤14, see Table 2). The right pallidum also showed a larger area of deflation in the medial posterior aspect of the pallidum for PwP compared to controls (see Figure 2). *PwP with and without cannabis use (PwP + C* vs. *PWP* − *C)*: The right and left putamen showed areas of enlargement in the PwP + C group on the medial surface which would abutt the pallidum (see Figure 3). There was a large area of enlargement on the lateral surface of the right putamen. The right thalamus was enlarged in the PwP + C group with a large cluster laterally (289 voxels) and at the site of the massa intermedia.

**TABLE 2:** Peak cluster co‐ordinates

	Cluster no	Size	Max *T*‐stat	*x*	*y*	*z*
*Primary analysis*: *Psychosis ± cannabis*						
**PwP vs. controls**						
R pallidum (increase in pts)	1	12	3.27	18	4	4
R pallidum (decrease in pts)	2	52	−4.16	16	−3	1
L amygdala (increase in pts)	3	14	2.83	−26	−6	−27
**PwP ± C vs PwP‐C**						
R thalamus (increase in PwP + C)	4	33	2.91	1	−17	6
R thalamus (increase in PwP + C)	5	289	2.89	14	−17	2
R putamen (increase in PwP + C)	6	22	3.17	19	7	1
R putamen (increase in PwP + C)	7	234	3.4	29	9	−5
L putamen (increase in PwP + C)	8	31	3.84	−22	3	3
**C ± C vs C‐C** None						
*Secondary analysis*: *Cannabis dependence*						
**All can‐dep ± versus can‐dep−**						
R thalamus (increase in Can‐Dep+)	1	1,422	3.81	19	−17	2
R putamen (increase in Can‐Dep+)	2	424	4.00	28	14	−5
R putamen (increase in Can‐Dep+)	3	84	3.17	15	10	−6
R putamen (increase in Can‐Dep+)	4	73	2.91	26	−2	−4
R amygdala (decrease in Can‐Dep+)	5	11	−3.10	18	0	−18
R amygdala (decrease in Can‐Dep+)	6	9	−3.60	15	−7	−20
R Accumbens (increase in Can‐Dep+)	7	3	3.12	9	12	−11
L putamen (increase in Can‐Dep+)	8	333	3.81	−29	9	−3
L putamen (increase in Can‐Dep+)	9	100	3.16	−21	−3	7
L putamen (increase in Can‐Dep+)	10	37	3.61	−20	4	−1
L amygdala (decrease in Can‐Dep+)	11	17	−3.01	−23	−2	−14

*Note:* Significant clusters after threshold free cluster enhancement, family wise error *p* < .05. Only significant surviving clusters shown. Co‐ordinates given in mm in MNI space.

*Volume*: In C + C the right accumbens was significantly larger than C − C Hedge's *g* 0.67, *p* = .046). This did not survive FDR correction. Otherwise there were no significant volumetric differences for any of the main comparisons (PwP vs. Controls; PwP + C vs. PwP − C and C + C vs. C − C) or for the secondary analysis (CanDep+ vs. CanDep−). Further volume data across groups are shown in Supporting Information.

## 4 DISCUSSION

Our main findings were that there was a bilateral increase in regional putamen shape bilaterally and in the right thalamus in PwP + C compared to PwP − C; whilst there was no evidence for selective regional shape deflation across the subcortical regions. There were no shape changes in controls with and without a history of cannabis use.

The putamen, alongside the caudate, together constitute the dorsal striatum which has been implicated in habit learning and compulsive behaviour (Everitt & Robbins, 2013). Increased putamen grey matter volume (Moreno‐Alcázar et al., 2018) and cerebral blood flow (Filbey, Aslan, Lu, & Peng, 2018) have been shown in healthy cannabis users compared to controls. Increased putamen volume has been shown to be associated with methamphetamine use (Andres et al., 2016; Chang et al., 2005; Jan, Lin, Miles, Kydd, & Russell, 2012), with alterations in putamen myeleoarchitecture in those at behavioural risk of addiction (Nord et al., 2019), and increased putamen volumes in probands with stimulant addiction and relatives of those with addictive disorder (Ersche et al., 2012). Two previous studies have examined cannabis use in the context of psychosis: one showed putamen volume increases in cannabis‐using compared to non‐using PwP (Koenders et al., 2015), whereas the other found neurochemical changes in the left putamen with higher NAA/choline ratio in cannabis‐using PwP compared to controls (Malchow et al., 2013). Our study thus extends their findings by demonstrating regional structural change using an alternative analytical strategy and confirming the finding of putamen enlargement in the PwP + C group.

We also find regional enlargement of the right thalamus in PwP + C compared to PwP − C. Consistent with our findings heavy cannabis users have been shown to have greater grey matter density in the right thalamus compared to non users (Matochik, Eldreth, Cadet, & Bolla, 2005), although volume decrease in cannabis users at risk for psychosis have also been reported (Kumra et al., 2012; Welch et al., 2011). The thalamus has a key role in the addiction circuitry and has been conceptualised to influence addiction pathology via cortico‐striatal‐thalamocortical loops (Huang, Mitchell, Haber, Alia‐Klein, & Goldstein, 2018). Thalamic nuclei have been demonstrated to regulate instrumental learning, behavioural flexibility and behavioural reinforcement (Bradfield, Hart, & Balleine, 2013; Lalive, Lien, Roseberry, Donahue, & Kreitzer, 2018; Marton, Seifikar, Luongo, Lee, & Sohal, 2018). Cortico–striatal–thalamocortical loops are particularly implicated in an array of psychopathology with compulsive behaviour including obsessive compulsive disorder and substance use (Fettes, Schulze, & Downar, 2017). In contemporary addiction models the dorsal striatal‐pallidal‐thalamic component of the circuit is particularly important in hardwiring habit formation the behavioural output driving compulsive use (Everitt & Robbins, 2005; Koob & Volkow, 2016). Taken together speculatively our findings of morphologically changes in the putamen and thalamus in PwP + C versus PwP – C, in substrates involved in habit formation, may represent a biomarker of compulsive use in this group. This may go some way to explaining why some PwP use cannabis despite clearly adverse consequences. In the absence of longitudinal analysis in this group, however, we are unable to disambiguate whether these changes were secondary to or pre‐existed the cannabis use.

Supporting this interpretation we show similar changes (bilateral putamen and right thalamic regional enlargements) in the whole sample in a secondary analysis when we reanalysed the data to compare lifetime cannabis dependence versus non dependence. However we are cautious of labelling these findings as a marker of substance dependence per se. This was not the main purpose of this study where we preferentially recruited a sample of PwP. We note a recent large study examining subcortical morphology found negative effects of alcohol dependence, positive effects of nicotine dependence and no main effect for other substance dependence (Chye et al., 2019b) and so limit our interpretations to those of a sample with early psychosis.

In PwP we find evidence that cannabis is associated with shape enlargement in structures involved with compulsive use. We do not find this in controls but do find increased volume (effect size 0.67) as compared to non‐users in the right nucleus accumbens (ventral striatum). In contemporary models both substrates serve different purposes in addiction circuits with the ventral striatum involved in mesolimbic reward and impulsive behaviour, and the dorsal striatum involved in habit formation and compulsive drug‐seeking (Everitt & Robbins, 2013; Koob & Volkow, 2010; Koob & Volkow, 2016). This raises the possibility of differing neurobiological alterations in cannabis‐using PwP versus otherwise healthy cannabis users. In our study the parameters of cannabis consumption including quantity, proportion of current users, and age of first use of cannabis did not significantly differ between cannabis using groups. Nevertheless, more individuals had a trend towards lifetime diagnosis of cannabis use disorder in the PwP + C group than the C + C group (79 vs. 50%). This is not unexpected given the association between cannabis dependence and psychosis (Fergusson, Horwood, & Swain‐Campbell, 2003; Wisdom, Manuel, & Drake, 2011) and may be a possible explanation for the neurobiological findings suggesting that the two cannabis using groups had been at differing stages of the addiction cycle. Different circuitry may explain why PwP use cannabis whilst in participants without psychotic disorder, psychotic experiences are associated with cessation of cannabis use (Sami, Notley, Kouimtsidis, Lynskey, & Bhattacharyya, 2019). In particular one could speculate that there is an increase in compulsivity in PwP compared to controls with a history of cannabis use, which may be associated with cannabis use disorder. Such a formulation could form the basis of hypothesis testing for further research though a multimodal approach.

We did not find evidence for regional deflation in cannabis‐using compared to non‐using PwP as we had particularly expected in the hippocampus. Reductions in volume and shape are considered to be consistent with an effect of regular cannabis use and are seen in chronic populations (Rais et al., 2008; Solowij et al., 2013). Longitudinal studies demonstrate that this reduction follows cannabis use (Rais et al., 2008; Rais et al., 2010; Welch et al., 2011; Welch, Stanfield, et al., 2011). This may support the view that the reduction in grey matter seen in long‐term users is a cumulative effect of cannabis use (Sami & Bhattacharyya, 2018).

There are several strengths in this study. We include four groups, including the control cannabis using group which has often been omitted from similar previous studies (Sami & Bhattacharyya, 2018). The T1‐weighted scans used are isotropic and relatively high resolution volumetric scans for addressing this question. We undertook segmentation using FSL FIRST which has been shown to be superior to FreeSurfer for putamen segmentation and comparable to gold standard expert manual tracing for basal ganglia structures (Perlaki et al., 2017). We covary for alcohol and tobacco use, as these can often confound findings and in line with recommendations from reviews in the field (Murray et al., 2017; Sami & Bhattacharyya, 2018). Furthermore, importantly the PwP + C and PwP – C patient groups are well matched across clinical parameters, including diagnosis functioning, symptomatology, days of hospitalisation and medication, indicating that any difference found cannot be accounted for by clinical differences. For shape analysis we used stringent criteria for multiple comparisons correction (TFCE, FWE < 0.5). FIRST undertakes analysis on a structure by structure basis, although it is arguable whether this would be any different if cluster correction was applied across the total number of subcortical surface vertices of all shapes.

Notwithstanding this we also note limitations to the study design. The study was cross‐sectional which precludes inference on the precise nature of causation between groups. We limited analysis to the subcortical deep grey matter structures and did not look at cortical thinning, cerebellar volumes, white matter tracts or ventricular enlargement, which may also be impacted by cannabis use. Furthermore the sample size was relatively modest to detect volumetric differences (Koenders et al., 2014) although comparable to previous such studies in shape (Smith et al., 2014; Smith et al., 2015; Solowij et al., 2013). For this reason, we did not try to disentangle gender‐specific effects, although there was no significant difference in gender between groups.

Taken together we have shown a neurobiological distinction between those in early psychosis with and without a history of cannabis use in the form of bilateral regional putamen enlargement. The putamen and thalamus may be key neurobiological substrates for compulsivity in the dual diagnosis group and further research should aim to delineate the behavioural and neurobiological correlates of these structures.

## CONFLICT OF INTEREST

None to declare.

## DATA AVAILABILITY STATEMENT

The data that support the findings of this study are available from the corresponding author upon reasonable request.

## Supporting information


**Table S1** Volumetric measures (mm^3^) of subcortical structures by group
**Figure S2** : Effect sizes of volumetric differences
