# Supplementary material for: Cannabis use in patients with early psychosis is associated with alterations in putamen and thalamic shape
Source: Hum Brain Mapp. 2020 Jul 20;41(15):4386–96. doi: 10.1002/hbm.25131 (PMC7502838; doi:10.1002/hbm.25131)
Supplement: Supplementary file 1 — Table S1 Volumetric measures (mm3) of subcortical structures by group Figure S2 : Effect sizes of volumetric differences [file HBM-41-4386-s001.docx]

**Supplementary Data**

**sTable 1: Volumetric measures (mm^3^) of subcortical structures by group:**

|  | ***Primary analysis*** | | | | | | | | ***Secondary analysis*** | | | |
| --- | --- | --- | --- | --- | --- | --- | --- | --- | --- | --- | --- | --- |
|  | **PwP+C**  *n=28* | | **PwP-C**  *n=26* | | **C+C**  *n=16* | | **C-C**  *n=22* | | **CanDep+**  *n=23* | | **CanDep-**  *n=69* | |
|  | Mean | *sd* | Mean | *sd* | Mean | *sd* | Mean | *sd* | Mean | *sd* | Mean | *sd* |
| Brainstem | 21695 | 1999 | 22241 | 2533 | 21445 | 1388 | 22179 | 2262 | 21481 | 1966 | 22069 | 2179 |
| Left accumbens | 614 | 108 | 594 | 119 | 646 | 82 | 591 | 123 | 630 | 108 | 601 | 112 |
| Right accumbens | 510 | 96 | 520 | 96 | 556 | 82 | 503 | 77 | 533 | 98 | 515 | 88 |
| Left amygdala | 1523 | 212 | 1567 | 214 | 1475 | 182 | 1489 | 280 | 1460 | 203 | 1539 | 230 |
| Right amygdala | 1505 | 231 | 1549 | 226 | 1501 | 207 | 1468 | 189 | 1447 | 237 | 1528 | 204 |
| Left caudate | 3972 | 488 | 3848 | 574 | 3841 | 388 | 3963 | 521 | 4046 | 519 | 3867 | 492 |
| Right caudate | 4086 | 529 | 3925 | 552 | 3951 | 401 | 4020 | 526 | 4119 | 557 | 3962 | 493 |
| Left hippocampus | 4080 | 416 | 4030 | 600 | 4003 | 334 | 4217 | 412 | 4046 | 458 | 4098 | 467 |
| Right hippocampus | 4048 | 429 | 4024 | 679 | 4091 | 327 | 4174 | 363 | 3991 | 455 | 4108 | 491 |
| Left pallidum | 1928 | 193 | 1893 | 185 | 1867 | 119 | 1899 | 99 | 1888 | 183 | 1904 | 152 |
| Right pallidum | 1934 | 163 | 1904 | 203 | 1907 | 86 | 1899 | 128 | 1916 | 138 | 1911 | 163 |
| Left putamen | 5608 | 658 | 5460 | 592 | 5349 | 380 | 5492 | 405 | 5581 | 627 | 5464 | 513 |
| Right putamen | 5503 | 634 | 5225 | 530 | 5203 | 340 | 5231 | 373 | 5444 | 546 | 5261 | 499 |
| Left thalamus | 8799 | 679 | 8700 | 908 | 8511 | 410 | 8773 | 775 | 8696 | 699 | 8721 | 751 |
| Right thalamus | 8587 | 693 | 8396 | 810 | 8398 | 398 | 8467 | 693 | 8522 | 733 | 8455 | 670 |

Legend: All volumes derived from FSL FIRST segmentation after normalisation; PwP+C: Early Psychosis with history of cannabis use; PwP-C: Early Psychosis without history of cannabis use: C+C Controls with history of Cannabis use; C-C: Controls without history of cannabis use; CanDep+: Participants with a history of cannabis dependence; CanDep-: Participants without a history of cannabis dependence; sd: standard deviations.

**sFig2: Effect Sizes of Volumetric differences**

Legend: Effect sizes based on Bias Corrected Hedge’s g. with 95% Confidence Intervals; PwP+C: Early Psychosis with history of cannabis use; PwP-C: Early Psychosis without history of cannabis use: C+C Controls with history of Cannabis use; C-C: Controls without history of cannabis use; Brstm: brainstem; L: left; R: right; accu: accumbens, amyg: amygdala; cau: caudate; hipp: hippocampus; pall: pallidum; puta: putamen; thal: thalamus.
